# Effects of Body Position and Hypovolemia on the Regional Distribution of Pulmonary Perfusion During One-Lung Ventilation in Endotoxemic Pigs

**DOI:** 10.3389/fphys.2021.717269

**Published:** 2021-09-10

**Authors:** Jakob Wittenstein, Martin Scharffenberg, Xi Ran, Yingying Zhang, Diana Keller, Sebastian Tauer, Raphael Theilen, Yusen Chai, Jorge Ferreira, Sabine Müller, Thomas Bluth, Thomas Kiss, Marcus J. Schultz, Patricia R. M. Rocco, Paolo Pelosi, Marcelo Gama de Abreu, Robert Huhle

**Affiliations:** ^1^Department of Anaesthesiology and Intensive Care Medicine, Pulmonary Engineering Group, University Hospital Carl Gustav Carus Dresden at Technische Universität Dresden, Dresden, Germany; ^2^Department of Intensive Care, Chongqing General Hospital, University of Chinese Academy of Science, Chongqing, China; ^3^Department of Anesthesiology, Affiliated Hospital of Southwest Medical University, Luzhou, China; ^4^Department of Anaesthesiology, Intensive-, Pain- and Palliative Care Medicine, Radebeul Hospital, Academic Hospital of the Technische Universität Dresden, Radebeul, Germany; ^5^Department of Intensive Care and Laboratory of Experimental Intensive Care and Anaesthesiology, Academic Medical Center, University of Amsterdam, Amsterdam, Netherlands; ^6^Laboratory of Pulmonary Investigation, Carlos Chagas Filho Institute of Biophysics, Federal University of Rio de Janeiro, Rio de Janeiro, Brazil; ^7^Department of Surgical Sciences and Integrated Diagnostics, University of Genoa, Genoa, Italy; ^8^Anesthesia and Critical Care, San Martino Policlinico Hospital, Istituto di Ricovero e Cura a Carattere Scientifico (IRCCS) for Oncology and Neurosciences, Genoa, Italy; ^9^Department of Intensive Care and Resuscitation, Anesthesiology Institute, Cleveland Clinic, Cleveland, OH, United States; ^10^Department of Outcomes Research, Anesthesiology Institute, Cleveland Clinic, Cleveland, OH, United States

**Keywords:** one-lung ventilation, OLV, pulmonary perfusion, thoracic anesthesia, hypovolemia, body position, HPV, gravity

## Abstract

**Background:** The incidence of hypoxemia during one-lung ventilation (OLV) is as high as 10%. It is also partially determined by the distribution of perfusion. During thoracic surgery, different body positions are used, such as the supine, semilateral, lateral, and prone positions, with such positions potentially influencing the distribution of perfusion. Furthermore, hypovolemia can impair hypoxic vasoconstriction. However, the effects of body position and hypovolemia on the distribution of perfusion remain poorly defined. We hypothesized that, during OLV, the relative perfusion of the ventilated lung is higher in the lateral decubitus position and that hypovolemia impairs the redistribution of pulmonary blood flow.

**Methods:** Sixteen juvenile pigs were anesthetized, mechanically ventilated, submitted to a right-sided thoracotomy, and randomly assigned to one of two groups: (1) intravascular normovolemia or (2) intravascular hypovolemia, as achieved by drawing ~25% of the estimated blood volume (*n* = 8/group). Furthermore, to mimic thoracic surgery inflammatory conditions, *Escherichia coli* lipopolysaccharide was continuously infused at 0.5 μg kg^−1^ h^−1^. Under left-sided OLV conditions, the animals were further randomized to one of the four sequences of supine, left semilateral, left lateral, and prone positioning. Measurements of pulmonary perfusion distribution with fluorescence-marked microspheres, ventilation distribution by electrical impedance tomography, and gas exchange were then performed during two-lung ventilation in a supine position and after 30 min in each position and intravascular volume status during OLV.

**Results:** During one-lung ventilation, the relative perfusion of the ventilated lung was higher in the lateral than the supine position. The relative perfusion of the non-ventilated lung was lower in the lateral than the supine and prone positions and in semilateral compared with the prone position. During OLV, the highest arterial partial pressure of oxygen/inspiratory fraction of oxygen (PaO_2_/*F*_I_*O*_2_) was achieved in the lateral position as compared with all the other positions. The distribution of perfusion, ventilation, and oxygenation did not differ significantly between normovolemia and hypovolemia.

**Conclusions:** During one-lung ventilation in endotoxemic pigs, the relative perfusion of the ventilated lung and oxygenation were higher in the lateral than in the supine position and not impaired by hypovolemia.

## Introduction

During one-lung ventilation (OLV), the incidence of relevant hypoxemia can be as high as 10% and can be associated with postoperative complications (Kazan et al., 2009). The incidence of hypoxemia is mainly determined by the pulmonary blood flow to the ventilated and non-ventilated lung, with the latter representing the intrapulmonary right-to-left shunt. During OLV, hypoxic pulmonary vasoconstriction (HPV) redirects pulmonary blood flow toward the ventilated lung. In turn, regional pulmonary blood flow is influenced by gravity (Szegedi et al., 2010), local mechanical forces (Alfery et al., 1981), and intravascular volume status (Deem et al., 1995), as summarized in the west-zone model (West et al., 1964). Furthermore, the atelectasis and hypo-ventilated zones and the hyper-inflated areas of the ventilated lung contribute to perfusion-ventilation mismatch and have an additive effect on shunting in the non-ventilated lung (Hedenstierna et al., 1986). Body position may further influence the distribution of pulmonary perfusion because of different gravitational and ventilation distributions in corresponding positions. Finally, the geometry of the vascular tree that branches asymmetrically plays an important role in the spatial distribution of pulmonary blood flow (Glenny and Robertson, 2011).

One-lung ventilation is required for different thoracic procedures to allow access to the surgical field. Depending on the surgical access, a patient can be placed in a supine, semilateral, lateral, or prone position. While the lateral decubitus position is most frequently used, the prone position is needed for certain esophageal and spinal surgery approaches. In addition, the supine position is required during mediastinal and cardiac surgery, and the semilateral position is used during open thoracic aortic repair (Crawford position). Currently, it is not known how these positions compare with respect to the distribution of regional pulmonary perfusion and gas exchange. Furthermore, during thoracic surgery, the incidence of major bleeding leading to acute intravascular hypovolemia can reach up to 5% (Schirren et al., 2015). It has been proposed that acute intravascular hypovolemia may alter hypoxic pulmonary vasoconstriction (Deem et al., 1995) and thereby gas exchange. However, the effect of hypovolemia on the distribution of pulmonary blood flow during OLV is not well-determined.

In this study, we aimed to determine the distribution of pulmonary blood flow during commonly used body positions for thoracic surgery during normo- and hypovolemia in pigs undergoing one-lung ventilation. We hypothesized that the pulmonary blood flow of the ventilated lung would be highest in the lateral decubitus position. We also hypothesized that intravascular hypovolemia impairs the redistribution of pulmonary blood flow because of an altered hypoxic pulmonary vasoconstriction.

## Methods

The Institutional Animal Care and Welfare Committee and the Government of the State of Saxony, Germany, approved the study (DD24.1-5131/449/71, TVV 69/2018). All the animals in this study received humane care in compliance with the Principles of Laboratory Animal Care formulated by the National Society for Medical Research and the US National Academy of Sciences Guide for the Care and Use of Laboratory Animals. This study also complied with the relevant aspects of the Animal Research: Reporting of *In Viv*o Experiments (ARRIVE) guidelines (Percie du Sert et al., 2020). The animals were kept at a controlled temperature and a light-dark cycle with free access to water and food.

### Experimental Protocol

**Figure 1:**
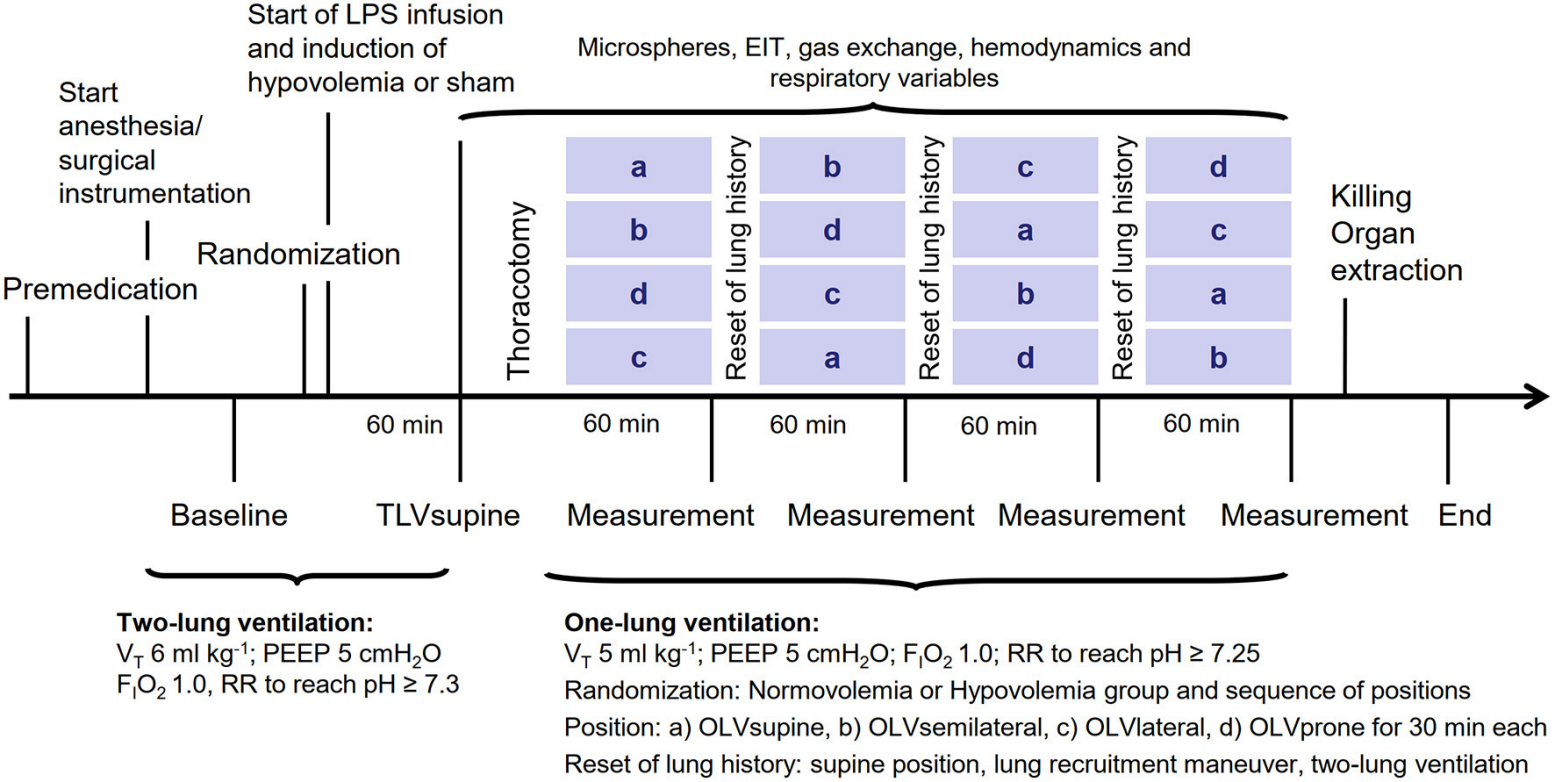
The time course of the interventions and measurements. LPS, lipopolysaccharide; V_T_, tidal volume; PEEP, positive end-expiratory pressure; F_I_O_2_, fraction of inspired oxygen; RR, respiratory rate; microspheres, measurement of regional pulmonary perfusion; EIT, electrical impedance tomography.

The time course of the experiments is presented in Figure 1. Sixteen female pigs (German landrace, weighing 35–49 kg, Danish Specific Pathogen Free Certification, www.spf.dk) were intramuscularly sedated with midazolam (1 mg kg^−1^) and ketamine (10 mg kg^−1^). Intravenous anesthesia was induced and maintained with midazolam (bolus of 0.5–1 mg kg^−1^, followed by 1 mg kg^−1^ h^−1^) and ketamine (bolus of 3–4 mg kg^−1^, followed by 15 mg kg^−1^ h^−1^). Muscle paralysis was achieved with atracurium (bolus 3–4 mg kg^−1^, followed by 3 mg kg^−1^ h^−1^). The intravascular volume was maintained with a crystalloid solution (E153; Serumwerk Bernburg AG, Bernburg, Germany) at a rate of 5 ml kg h^−1^. The mean arterial pressure was kept >60 mmHg by norepinephrine and colloid infusion, as appropriate. Colloids were used in the case of increasing hemoglobin. Furthermore, the animals were ventilated in a volume-controlled mode: a fraction of inspired oxygen (F_I_O_2_) of 1, a tidal volume (V_T_) of 6 ml kg^−1^, a positive end-expiratory pressure (PEEP) of 5 cm H_2_O, an inspiratory: expiratory (I:E) ratio of 1:1, a constant gas flow of 25 L/min, and a respiratory rate (RR) adjusted to arterial pH >7.3.

All skin incisions were preceded by the infiltration of 2–5 ml lidocaine 2%. After the surgical preparation of the right internal carotid artery, a pulse contour cardiac output (PiCCO) catheter (20 cm; Pulsion Medical Systems SE, Feldkirchen, Germany) was inserted to continuously monitor the arterial pressure. A 7.5 Fr. pulmonary artery catheter (Opticath; Abbott, Abbott Park, IL, United States) was used to measure cardiac output (CO), and pulmonary artery pressure was advanced through an 8.5 Fr. sheath placed in the right external jugular vein until typical pulmonary arterial pressure waveforms were observed. Urine was collected with a bladder catheter inserted through a median mini-laparotomy. For lung separation, a left-sided double-lumen tube (39 Fr., Silbroncho Fuji, Tokyo, Japan) was placed through a tracheotomy, where the bronchial tip was inserted into the left main bronchus, under fiberoptic control conditions (AmbuaScope 3 and AmbuaView, Ambu GmbH, Bad Nauheim, Germany). In another investigation, the results of which are being published elsewhere, a left-sided video-assisted thoracoscopy was performed, where three pressure sensors were attached to the parietal pleura in the left hemi-thorax, as described previously (Kiss et al., 2019). Thereafter, the baseline measurements of gas exchange, respiratory signals, hemodynamics, and the distribution of ventilation by electrical impedance tomography (EIT) were performed (baseline).

The animals were randomly assigned to normovolemia or hypovolemia. For the induction of moderate hypovolemia, 25% of the calculated blood volume, estimated as 70 ml kg^−1^ (Hannon et al., 1990), was drawn from the central venous catheter. To mimic the inflammatory response to surgical trauma due to major thoracic surgery (Takenaka et al., 2006; Sánchez-Pedrosa et al., 2018), 0.5 μg/kg/h of lipopolysaccharides (LPS) from *E. coli* O111:B4 (Sigma-Aldrich, St. Louis, MO, United States) was continuously infused through the central venous line. In previous studies, a low-dose LPS infusion was shown to reduce hypoxic pulmonary vasoconstriction (Reeves and Grover, 1974; Theissen et al., 1991). An hour after the start of LPS infusion, the two-lung ventilation supine (TLVsupine) measurements of regional pulmonary perfusion (microspheres), gas exchange, respiratory signals, hemodynamics, and the distribution of ventilation were performed. Thereafter, the animals were randomly submitted to one of four sequences during OLV according to a Latin square design, namely, (1) a-b-c-d, (2) b-d-a-c, (3) d-c-b-a, and (4) c-a-d-b (30 min per body position, crossover), with a = supine, b = left semilateral, c = left lateral, and d = prone position. To mimic a thoracic surgery, a right-sided thoracotomy was performed between the medial-clavicular and anterior axillary lines in the 4th−5th intercostal space by placing a rib spreader. For OLV, a volume-controlled mode was used: V_T_ of 5 ml kg^−1^, F_I_O_2_ of 1, PEEP of 5 cmH_2_O, I:E of 1:1, and RR of 30–35 min^−1^ titrated to achieve an arterial pH of >7.25 and a flow of 25 L min^−1^. Thirty minutes after placing the animals in the respective body positions (OLVsupine, OLVsemilateral, OLVlateral, OLVprone), the measurements of regional pulmonary perfusion, gas exchange, respiratory signals, hemodynamics, and the distribution of ventilation were performed. To reset lung history between interventions, the animals were placed in the supine position and disconnected from the ventilator. An alveolar recruitment maneuver was then performed, with two-lung ventilation resuming thereafter until the normalization of gas exchange, before the start of each position.

### Measurements

#### Measurement of Regional Pulmonary Blood Flow

The distribution of regional pulmonary blood flow was marked with IV-administered fluorescence and color-labeled 15-μm diameter microspheres (Thermo Fisher Scientific, Waltham, MA, United States). The colors used were blue, blue-green, yellow-green, orange, and red. To avoid bias, the colors were randomly assigned at any given time point. Immediately before injection, the microspheres were vortexed, sonicated for 90 s, and drawn into 2-ml syringes. All the injections were performed for over 60 s to average the blood flow over several cardiac and respiratory cycles. During the injection, ~1.5 × 10^6^ microspheres were administered.

Postmortem lungs were extracted *en bloc* and flushed with 50 ml kg^−1^ of a hydroxyethyl starch 130/0.4 solution (Voluven, Fresenius Kabi, Bad Homburg, Germany) and air-dried by continuous tracheal airflow for 7 days with a continuous pressure of 25 cm H_2_O. The lungs were then coated with a one-component polyurethane foam (BTI Befestigungstechnik, Ingelfingen, Germany), suspended vertically in a square box, and embedded in a rapidly setting urethane foam (polyol and isocyanate; Elastogran, Lemförde, Germany).

The foam block was then cut into cubes of ~1.2 cm^3^. Each cube was weighed and assigned a three-dimensional coordinate. The samples were then soaked for 7 days in 3 ml of 2-ethoxyethyl acetate (Aldrich Chemical Co. LLC, Milwaukee, WI, United States) to retrieve the fluorescent dye. The fluorescence was read in a luminescence spectrophotometer (LS-50B; Perkin-Elmer, Beaconsfield, United Kingdom), with the measured intensity of fluorescence in each probe then being normalized according to its weight (x_i_). The relative pulmonary blood flow Q_rel, i_ of a probe i was also determined according to:


$$\begin{array}{l} Q_{rel,i}=\frac{x_{i}}{\frac{1}{n}\sum x_{i}} \end{array}$$

with the denominator holding the mean relative blood flow of one lung per time point. The mean normalized relative blood flow was, therefore, 1.

The distributions of pulmonary blood flow along the craniocaudal, ventrodorsal, and left-right axes under each of the experimental conditions were assessed by linear regression. Additionally, a three-dimensional reconstruction of the lung was performed, considering the spatial coordinates of each lung piece and the pulmonary blood flow at each of the x (left-right), y (dorsal-ventral), and z (caudal-cranial) coordinates. Color mapping was performed to identify the regional distribution of pulmonary blood flow based on Q_rel, i_. The color map was then normalized by the maximum Q_rel_ under each of the experimental conditions, resulting in a color scale ranging from white (0, lowest perfusion) to red (1, highest perfusion). The relative centers of perfusion along the left-right axes, the dorsal-ventral, and the caudal-cranial axis were calculated by.


$$\begin{array}{l} CoP_{x}=\frac{1}{\sum Q_{rel,i}\left(x,y,z\right)}\overset{1}{\underset{x=0}{\sum }}x\cdot \underset{y,z}{\sum }Q_{rel.i}\left(x,y,z\right) \end{array}$$

with the three body directions represented by x, y, and z. The coefficient of variation (ratio of the SD to the mean in percent) of mean pulmonary perfusion was calculated to determine the spatial heterogeneity of pulmonary perfusion distribution.

#### Electrical Impedance Tomography

Electrical impedance tomography measurements were conducted with an operating frequency of 130 kHz and 50 frames s^−1^. Raw measured EIT data were then filtered at 50 Hz and reconstructed using PulmoVista® 500 (Drägerwerk AG & Co. KGaA, Lubeck, Germany), a commercially available software. Each EIT image of the resulting reconstructed temporal image series consisted of 32 × 32 pi. The reconstruction of these images was carried out as described in detail by the group of authors of this study (Bluth et al., 2019). The global region of interest was a half-sphere covering the left hemisphere of the EIT, thus, only containing the ventilated lung, as described previously (Wittenstein et al., 2020). The center of ventilation was defined as the median of tidal impedance changes (surrogate for ventilation) along the dorsoventral axis and left-right axis of the left lung and expressed as a percentage, with 0% representing most dorsal and most left and 100% most ventral and most central lung zones.

#### Gas Exchange and Hemodynamics

Arterial and mixed venous blood samples were analyzed using a blood gas analyzer (ABL 80 Flex Basic, Radiometer Medical, Copenhagen, Denmark). The mean arterial and pulmonary artery pressures were measured continuously, and cardiac output was determined with a pulmonary artery catheter using a conventional thermodilution method. Extravascular lung water (EVLW, a surrogate for lung injury), intrathoracic blood volume (ITBV), and global end-diastolic blood volume (GEDV, a surrogate for cardiac preload), systemic vascular resistance (SVR, a surrogate for cardiac afterload), and stroke volume (SV) were determined using the PiCCO catheter. The values were then normalized to body surface area [pulmonary vascular resistance index (PVRI), systemic vascular resistance index (SVRI), global end-diastolic volume index (GEDVI), and intrathoracic blood volume index (ITBVI), respectively] and body weight [extravascular lung water index (EVLWI]), as reported previously (Kelley et al., 1973). Furthermore, PiCCO was not used to guide fluid treatment, since the normal values of pigs lie outside the reference ranges for humans (Längin et al., 2020).

#### Respiratory Signals

Airway flow was measured with the internal sensors of the ventilator. On the other hand, airway pressure was measured at the y-piece with a custom-made measurement system composed of a pressure transducer (163PC01D48-PCB; FirstSensors AG, Berlin, Germany) and corresponding hardware and software for amplification and recording (custom-built software written in LabVIEW, National Instruments, Austin, TX, United States). Furthermore, respiratory system elastance (E_RS_) and resistance (R_RS_) were determined by the multiple linear regression of the linear equation of motion composed of the R_RS_ and E_RS_ two-compartmental model of the respiratory system.

### Statistical Analyses

Sample size calculation was based on the perfusion measurements of relative perfusion distribution by positron-emission tomography using ^68^Ga-labeled microspheres from a previous study of the group of authors on pigs under two-lung ventilation conditions and different levels of PEEP (Bluth et al., 2019). In this study, we expected that the relative perfusion of the ventilated lung during OLV would be higher than during TLV. Assuming an effect size of 2, we estimated that eight animals per group would yield a power of 80% to detect the difference in the distributions of pulmonary perfusion between TLV and OLV in the supine, semilateral, lateral, and prone positions, with α = 0.01 corrected for multiple comparisons. The data were presented as mean and SD if not stated otherwise. The statistical analysis was conducted with SPSS (Version 27, IBM Corp., Armonk, NY, United States). Significance was accepted at *P* < 0.05. The differences between the two groups, respective body positions, and the sequences of interventions were compared using a linear mixed-effects model with repeated measures, using composite ventilation-position (levels: TLVsupine, OLVsupine, OLVsemilateral, OLVlateral, and OLVprone) as the within-subject factor and with group and sequence as between subject-factors. The significance of the within-subject factors was corrected for sphericity according to Greenhouse–Geisser. Pairwise *post-hoc* multiple comparisons were also performed according to least significant difference (LSD) when appropriate.

## Results

### Characteristics of Animal and Experimental Protocol

Body weight, total time of anesthesia, total time on mechanical ventilation, the cumulative doses of crystalloids and colloids, and total urine output did not differ significantly between normovolemia and hypovolemia, while the cumulative norepinephrine dose was higher in hypovolemia than in normovolemia (Table 1). Hemoglobin was not different between the groups (*P* = 0.593). In the hypovolemia group, 755 ± 80 ml of blood was drawn (Table 1), resulting in a significant decrease in ITBVI (baseline: 765 ± 79 ml m^−2^ vs. TLVsupine: 619 ± 122 ml m^−2^; *P* = 0.006) and GEDVI (baseline: 612 ± 63 ml m^−2^ vs. TLVsupine: 496 ± 98 ml m^−2^; *P* = 0.006) at TLVsupine *vs*. baseline. In all the animals, the LPS infusion resulted in a significant increase in PVRI at TLVsupine vs. baseline (baseline: 150 ± 53 dyn s cm^−5^ m^−2^; TLVsupine: 365 ± 243 dyn s cm^−5^ m^−2^; *P* = 0.006) and SVRI (baseline: 1,153 ± 237 dyn s cm^−5^ m^−2^; TLVsupine: 1,602 ± 579 dyn s cm^−5^ m^−2^; *P* = 0.014).

**Table 1:** Characteristics of the animal and experimental protocol.

**Variable**	**Normovolemia**	**Hypovolemia**	***P* =**
Body weight [kg]	43.5 ± 1.4	43.1 ± 4.6	0.803
Total anesthesia time [min]	713 ± 52	738 ± 81	0.493
LPS total dose [μg]	178.6 ± 13.6	187.2 ± 25.8	0.422
Aspirated blood volume [ml]	0 ± 0	755 ± 80	≤ 0.001
Crystalloid infusion [ml kg^−1^]	47 ± 6	46 ± 10	0.847
Colloids [mlkg^−1^]	7 ± 6	8 ± 8	0.843
Norepinephrine [μg kg^−1^]	3 ± 5	52 ± 53	0.036
Cumulative urine output [mL]	945 ± 489	809 ± 303	0.514

*Mean ± SD; significance was accepted at P < 0.05. Differences between the two groups were compared by Student's t-test*.

### Regional Pulmonary Blood Flow (Primary Endpoint)

**Figure 2:**
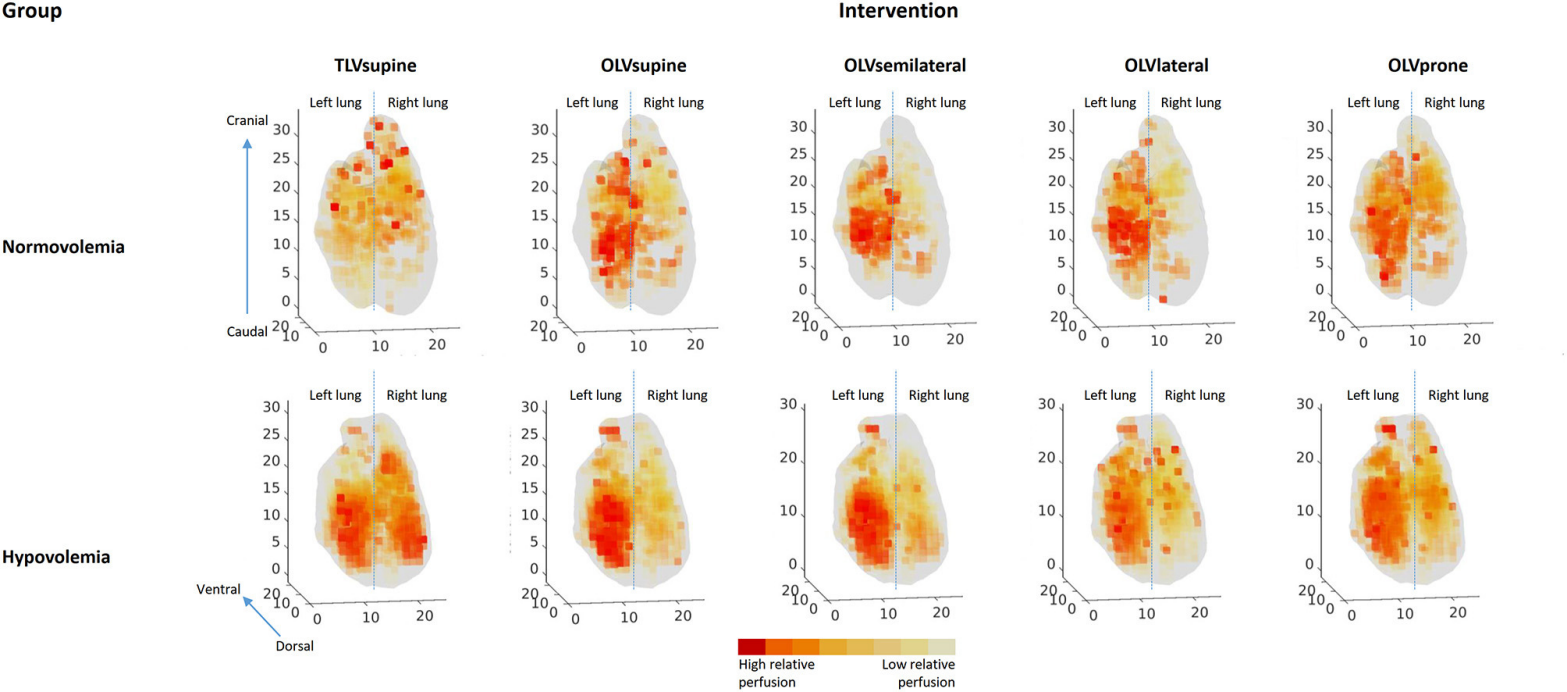
Representative three-dimensional perfusion maps for one animal from each group. Lungs are shown from the dorsal with the left lung on the left side and right lung on the right side. Axes are divided in cm. Lighter colors represent lower relative pulmonary perfusions, while darker colors represent higher relative pulmonary perfusions.

**Figure 3:**
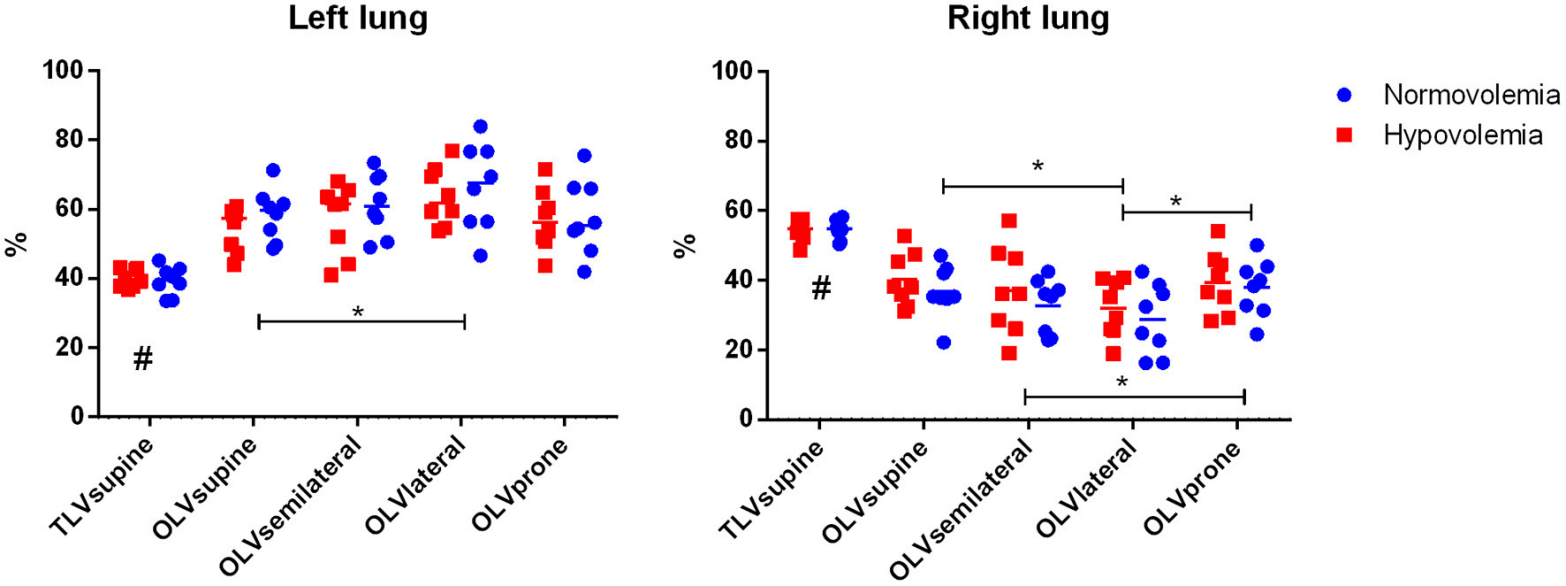
Relative perfusion of the left and right lungs. Mean and single values. Significance was accepted at *P* < 0.05. Differences between the two groups, respective body positions, and the sequences of interventions were compared using a linear mixed-effects model with repeated measures, with TLVsupine, OLVsupine, OLVsemilateral, OLVlateral, and OLVprone as within-subject factors and with group and sequence as fixed between subject-factors. The significance of the within-subject factors was corrected for sphericity according to Greenhouse–Geisser. Pairwise *post-hoc* multiple comparisons were performed according to LSD when appropriate. #*P* < 0.05 TLVsupine vs. all the others, *P < 0.05. The relative perfusions of the ventilated and the non-ventilated lungs were not different between normo- and hypovolemia (*P* = 0.457 and *P* = 0.418, respectively). Mixed effects position × group ventilated lung: *P* = 0.852 and mixed effects position × group non-ventilated lung: *P* = 0.891.

Compared with TLVsupine, the OLV resulted in the shift of perfusion toward the ventilated left lung, irrespective of position (Figures 2, 3, Table 2). During OLV, the relative perfusion of the ventilated lung was higher in the lateral as compared with the supine position, while the relative perfusion of the non-ventilated lung was lower in the lateral position as compared with the supine and prone positions, and in the semilateral compared with the prone position. The relative perfusions of the ventilated and non-ventilated lungs were not different between normovolemia and hypovolemia.

**Table 2:** Perfusion of the left lung.

**Variable**	**Group**	**TLVsupine**		**OLVsupine**		**OLVsemilateral**		**OLVlateral**		**OLVprone**		**Sequence *P* =**	**Group *P* =**	**Position *P* =**	**ME *P* =**
Center of perfusion left-right axis [% from left]	Normo	30.7 ± 5.0	p	28.8 ± 3.6	se, l, p	31.6 ± 2.6	p	31.1 ± 4.2	p	32.8 ± 2.6		0.614	0.695	≤ 0.001	0.368
	Hypo	28.5 ± 4.9		29.0 ± 3.3		29.9 ± 3.5		30.8 ± 3.1		33.4 ± 2.7					
Center of perfusion dorsal-ventral axis [% from dorsal]	Normo Hypo	27.5 ± 3.6 27.5 ± 4.4		28.9 ± 3.0 28.3 ± 3.9	sl, l, p	27.1 ± 3.1 27.7 ± 4.1		27.3 ± 2.9 27.5 ± 3.3		27.5 ± 3.0 27.7 ± 3.8		0.673	0.948	0.025	0.627
Center of perfusion caudal-cranial axis [% from caudal]	Normo Hypo	45.4 ± 6.0 42.9 ± 3.7	sl	46.0 ± 5.2 44.2 ± 4.1		46.8 ± 4.6 46.1 ± 4.5		46.1 ± 4.7 45.0 ± 4.0		45.2 ± 3.1 45.3 ± 3.5		0127	0.462	0.048	0.444
CV of relative perfusion %	Normo	157.9 ± 19.1	sl, l, p	154.4 ± 11.2		151.4 ± 15.9		147.2 ± 10.3		139.4 ± 10.4	s, se	0.026	0.075	≤ 0.001	0.104
	Hypo	180.0 ± 24.3		153.4 ± 14.7		152.9 ± 14.9		145.4 ± 8.4		143.8 ± 8.0					

*Mean ± SD; Normo, normovolemia group; Hypo, hypovolemia group; TLV, two-lung ventilation; OLV, one-lung ventilation; CV, coefficient of variation; ME, mixed effects position × group. Significance was accepted at P < 0.05. The differences between the two groups, respective body positions, and the sequences of interventions were compared using a linear mixed-effects model with repeated measures, with TLVsupine, OLVsupine, OLVsemilateral, OLVlateral, and OLVprone as within-subject factors and with group and sequence as fixed between subject-factors. The significance of the within-subject factors was corrected for sphericity according to Greenhouse–Geisser. Pairwise post-hoc multiple comparisons were performed according to least significant difference (LSD) when appropriate. s P < 0.05 vs. OLVsupine, se P < 0.05 vs. OLVsemilateral, l P < 0.05 vs. OLVlateral, and p P < 0.05 vs. OLVprone*.

In the ventilated left lung, the center of relative perfusion along the left-right axis shifted toward the hilum during OLVsemilateral, OLVlateral, and OLVprone as compared with OLVsupine. Along the dorsal-ventral axis, the perfusion shifted toward the dorsal during OLVsemilateral, OLVlateral, and OLVprone as compared with OLVsupine. Along the caudal-cranial axis, the perfusion shifted toward the cranial in the semilateral position as compared with TLVsupine. The center of perfusion did not differ between normovolemia and hypovolemia (Table 2).

The spatial heterogeneity of relative perfusion in the ventilated left lung was highest during TLVsupine and lowest during OLV in the prone position, while there was no difference between normovolemia and hypovolemia (Table 2).

### Regional Ventilation (EIT)

In the ventilated left lung, the center of ventilation along the left-right axis shifted toward the lung hilum during OLV in all positions as compared with TLVsupine. Furthermore, it also shifted toward the lung hilum during OLV in the prone as compared with the lateral position (Table 3). In addition, the center of ventilation along the dorsal-ventral axis of the left lung shifted toward the dorsal during OLV in the prone as compared with the supine position. The center of ventilation along the left-right and dorsoventral axes did not differ among the groups (Table 3).

**Table 3:** Electrical impedance tomography of the left lung.

**Variable**	**Group**	**TLVsupine**		**OLVsupine**		**OLVsemilateral**		**OLVlateral**		**OLVprone**		**Sequence *P* =**	**Group *P* =**	**Position *P* =**	**ME *P* =**
CoVleftright [% from left)	Normo	51.1 ± 3.9	s, se, l, p	50 ± 3.6		56.5 ± 4.3		55 ± 3.7	p	54.8 ± 3.4		0.527	0.456	≤ 0.001	0.743
	Hypo	51.6 ± 3.2		51.8 ± 3.4		56.5 ± 1.8		56.3 ± 2.3		54.3 ± 3.6					
CoVdorso-ventral [% from dorsal]	Normo	49.6 ± 1.9		49.2 ± 1.6	p	49.8 ± 1.5		49.6 ± 1.4		48.9 ± 1.4		0.822	0.636	0.040	0.958
	Hypo	49.1 ± 2.2		49.3 ± 1.6		50 ± 1.2		50 ± 1		49.4 ± 1.8					

*Mean ± SD; Normo, normovolemia group; Hypo, hypovolemia group; TLV, two-lung ventilation; OLV, one-lung ventilation; CoV, center of ventilation along the left-right and dorso-ventral axes; ME, mixed effects position × group. Significance was accepted at P < 0.05. Differences between the two groups, respective body positions, and the sequences of interventions were compared using a linear mixed-effects model with repeated measures, with TLVsupine, OLVsupine, OLVsemilateral, OLVlateral, and OLVprone as within-subject factors and with group and sequence as fixed between subject factors. The significance of the within-subject factors was corrected for sphericity according to Greenhouse–Geisser. Pairwise post-hoc multiple comparisons were performed according to LSD when appropriate. s P < 0.05 vs. OLVsupine, se P < 0.05 vs. OLVsemilateral, l P < 0.05 vs. OLVlateral, and p P < 0.05 vs. OLVprone*.

### Gas Exchange

The variables of gas exchange are summarized in Table 4, where PaO_2_/F_I_O_2_ differed significantly between TLVsupine and OLV in all the positions. It was higher in the lateral as compared with the other positions during OLV, while there was no difference between normovolemia and hypovolemia. Additionally, PaCO_2_ in the arterial blood gas analysis differed significantly between TLVsupine and OLV in all the positions, while it was not different for the different positions during OLV, and there was no group difference. Arterial pH also differed significantly between TLVsupine and OLV in all the positions, while it was similar in the different positions during OLV. Arterial pH was lower in hypovolemic as compared with normovolemic animals. Mixed venous oxygen saturation was significantly different between TLVsupine and OLVsupine and between OLVsemilateral and OLVprone. Furthermore, the mixed venous oxygen saturation was higher during OLVlateral compared with OLVsupine and OLVprone. There was no difference between the two groups.

**Table 4:** Gas exchange.

**Variable**	**Group**	**BL**	**TLVsupine**		**OLVsupine**		**OLVsemilateral**		**OLVlateral**		**OLVprone**		**Sequence *P* =**	**Group *P* =**	**Position *P* =**	**ME *P* =**
pHa	Normo	7.38 ± 0.04	7.39 ± 0.03	s, se, l, p	7.29 ± 0.08	l	7.28 ± 0.1		7.31 ± 0.08		7.29 ± 0.08		0.481	0.304	≤ 0.001	0.629
	Hypo	7.42 ± 0.07	7.39 ± 0.06		7.23 ± 0.13		7.25 ± 0.11		7.26 ± 0.12		7.25 ± 0.11					
PaCO_2_ [mmHg]	Normo	54 ± 6	53 ± 5	s, se, l, p	65 ± 13		66 ± 12		63 ± 11		64 ± 13		0.464	0.425	≤ 0.001	0.273
	Hypo	50 ± 12	51 ± 7		72 ± 17		69 ± 13		69 ± 15		69 ± 15					
F_I_O_2_/PaO_2_ [mmHg]	Normo	509 ± 50	562 ± 45	s, se, l, p	78 ± 18		89 ± 20		123 ± 45	s, se, p	78 ± 9		0.936	0.878	≤ 0.001	0.940
	Hypo	504 ± 105	565 ± 64		85 ± 26		80 ± 24		101 ± 14		94 ± 24					
SaO_2_ [%]	Normo	100 ± 1	99 ± 4	s, se, l, p	90 ± 7		91 ± 6		95 ± 5	s	91 ± 8		0.862	0.578	≤ 0.001	0.603
	Hypo	100 ± 1	100 ± 0		85 ± 10		90 ± 9		94 ± 5		89 ± 8					
SvO_2_ [%]	Normo	77 ± 7	78 ± 12	s, se, p	64 ± 9		64 ± 10		76 ± 9	s, p	65 ± 13		0.862	0.578	≤ 0.001	0.459
	Hypo	78 ± 6	79 ± 6		62 ± 14		65 ± 12		67 ± 12		61 ± 16					

*Mean ± SD; Normo, normovolemia group; Hypo, hypovolemia group; BL, baseline; TLV, two-lung ventilation; OLV, one-lung ventilation; pHa, arterial pH, F_I_O_2_, fraction of inspired oxygen; PaO_2_, arterial partial pressure of oxygen; PaCO_2_, arterial partial pressure of carbon dioxide, SaO_2_, arterial oxygen saturation; SvO_2_, mixed venous oxygen saturation; ME, mixed effects position × group. Significance was accepted at P < 0.05. Differences between the two groups, respective body positions, and the sequences of interventions were compared using a linear mixed-effects model with repeated measures, with TLVsupine, OLVsupine, OLVsemilateral, OLVlateral, and OLVprone as within-subject factors and with group and sequence as fixed between subject-factors. The significance of the within-subject factors was corrected for sphericity according to Greenhouse–Geisser. Pairwise post-hoc multiple comparisons were performed according to LSD when appropriate. s P < 0.05 vs. OLVsupine, se P < 0.05 vs. OLVsemilateral, l P < 0.05 vs. OLVlateral, and p P < 0.05 vs. OLVprone*.

### Hemodynamic and Respiratory Variables

Hemodynamic and respiratory variables are summarized in the (Supplementary Material Tables 1, 2).

## Discussion

In a model of thoracic surgery and OLV with normovolemia and moderate hypovolemia in pigs, we found that (1) the relative pulmonary blood flow of the ventilated lung was highest in the lateral position and lowest in the supine position; (2) the relative pulmonary blood flow of the non-ventilated lung was lowest in the lateral position and highest in the supine and prone positions; (3) the spatial heterogeneity of pulmonary blood flow of the ventilated lung was lowest in the prone position; (4) PaO_2_/F_I_O_2_ during OLV was highest in the lateral position; and (5) hypovolemia did not influence the distribution of perfusion, irrespective of body position.

To the knowledge of the authors, this is the first *in vivo* study that systematically investigated the effects of body position and intravascular volume status on the distribution of relative pulmonary perfusion during OLV in a clinically relevant model of thoracic surgery. Previous physiological studies have investigated the effects of OLV through either a closed chest or minor surgeries (Bardoczky et al., 2000; Szegedi et al., 2010). In contrast, major surgeries trigger the inflammatory cascade (Hannon et al., 1990; Kiss et al., 2019), which can blunt HPV (Himmat et al., 2018) and interfere with the distribution of pulmonary perfusion. We also used LPSs to mimic the inflammatory response to major thoracic surgery, which was previously shown to reliably reduce hypoxic pulmonary vasoconstriction (Reeves and Grover, 1974; Theissen et al., 1991) while not altering hemodynamics significantly (Traber et al., 1989). Another strength of this study is that normovolemia and moderate hypovolemia (Silva et al., 2013), both of which may occur during thoracic surgery (Nakamura et al., 2015) and can influence HPV (Deem et al., 1995), were addressed. We chose the left semilateral and lateral decubitus positions because of the fact that the effects of mediastinal compression are more pronounced in the left than in the right semilateral and lateral positions (Chang et al., 2002).

### Effects of Body Position on Regional Pulmonary Perfusion and Ventilation

The finding that the relative perfusion of the ventilated lung was highest in the lateral decubitus position during OLV is in line with clinical data (Bardoczky et al., 2000; Szegedi et al., 2010). In the lateral decubitus position, gravitational forces, in addition to HPV, reduce the blood flow of the non-ventilated lung. Furthermore, HPV also allows ventilation-perfusion matching by reducing perfusion to poorly oxygenated lung tissue through smooth muscle contractions in primarily low-resistance pulmonary arteries (Weir et al., 2005). In addition to HPV, hypercapnic pulmonary vasoconstriction (HCPV) reduces perfusion to hypo-ventilated and, therefore, hypercapnic lung regions (Dorrington et al., 2010). In the supine and prone positions, HPV, HCPV, and regional mechanical forces determine regional pulmonary perfusion, while gravity does not influence the shift of perfusion toward the ventilated lung (Szegedi et al., 2010). In addition to the geometry of the vascular tree, which branches asymmetrically (Glenny and Robertson, 2011) regional mechanical forces in the ventilated lung determine the distribution of pulmonary blood flow. Regional mechanical forces are defined by tissue deformation, thorax shape (Tawhai et al., 2009), and mechanical ventilation (Alfery et al., 1981). High airway pressures can divert the blood flow from the ventilated to the non-ventilated lung by compressing the capillaries. In fact, peak, mean, and plateau airway pressures were higher during OLVsupine as compared with OLVlateral, possibly contributing to lower relative perfusion of the ventilated lung in this position.

HPV, HCPV, gravity, and regional mechanical forces as well as lung volume do not only influence the distribution of blood flow toward the ventilated and non-ventilated lung but also determine its regional distribution within the ventilated lung. In fact, during OLV, the lateral position center of perfusion along the left-right axis shifted toward the lung hilum as compared with the supine position, while the center of ventilation along the left-right axis shifted toward peripheral lung areas as compared with the supine position, possibly resulting in improved ventilation-perfusion matching. This might represent another mechanism of better gas exchange for this body position during OLV.

During OLV in the semilateral position, HPV is augmented by gravity. In line with our results, in a small clinical trial arterial saturation as a surrogate for regional pulmonary perfusion was not different between semilateral and lateral position (Watanabe et al., 2000). Thirty-three adult patients undergoing right thoracotomy with left OLV were divided into three groups: supine position (*n* = 11), left semilateral decubitus position (*n* = 9), and left lateral decubitus position (*n* = 13). The final PaO_2_ and SaO_2_ at the end of the OLV were lowest in the supine position, while there was no difference between the semilateral and lateral decubitus positions (Watanabe et al., 2000).

### Gas Exchange

The fact that PaO_2_/F_I_O_2_ was higher during OLV in lateral as compared with the supine, semilateral, and prone positions can be explained by the differences in regional pulmonary perfusion, namely, the lower perfusion of the non-ventilated lung and the better ventilation-perfusion matching of the ventilated lung. The gas exchange itself, especially hypercapnia, may influence intrapulmonary shunt and HPV (Benumof et al., 1976). However, PaCO_2_ did not differ significantly during OLV in the four positions and the groups. Nevertheless, arterial pH was lower in the hypovolemia group. Furthermore, lower arterial pH has the potential to increase HPV (Brimioulle et al., 1990). In this study, the lower arterial pH in the hypovolemia group might have counteracted the deleterious effects of hypovolemia on HPV, resulting in similar regional perfusions for both groups. However, the differences in the arterial pH between the groups were small and most likely clinically irrelevant.

### Intravascular Volume Status

The finding rejects the hypothesis that intravascular hypovolemia influences regional pulmonary perfusion, which is in contrast with previous studies. In isolated rat lungs perfused with plasma, HPV was weakened, when compared with lungs perfused with blood (McMurtry et al., 1977; Deem et al., 1998). Similar results were found in isolated rat, cat, and rabbit lungs (Hakim and Malik, 1988). In this experiment, we performed low-dose LPS infusions in both groups, which altered pulmonary vascular resistance (Theissen et al., 1991), possibly masking the further effects of acute intravascular hypovolemia on the distribution of pulmonary perfusion. As stated, during OLV in the supine and prone positions, the distribution of regional perfusion to the ventilated and non-ventilated lungs is mainly determined with HPV, HCPV, and lung volume, with gravity playing a minor role. Therefore, the most pronounced effects of acute intravascular hypovolemia would be expected in these positions. However, even in OLVsupine and OLVprone, we found no differences between the groups, supporting the claim that an acute moderate hemorrhage during thoracic surgery only has a minor effect on the distribution of pulmonary perfusion.

### Possible Clinical Implications

The results suggest that the lateral decubitus position may serve as a means to improve the distribution of perfusion and oxygenation during OLV. In fact, during OLV in the supine, semilateral, and prone positions, but not the lateral decubitus position, the central venous oxygen saturation was lower than 70%, indicating tissue hypoxia (Sevuk et al., 2016) in the normovolemia group. Interestingly, moderate hypovolemia did not influence the distribution of perfusion and shunting, challenging the concept that the intravascular volume expansion in patients with volume depletion might be useful for the reversal of hypoxemia during OLV.

### Limitations

This study has several limitations. First, the thoracic surgery model did not fully represent the clinical scenario, especially because of the lack of the surgical manipulation of the lungs and its potential effects on atelectasis in the dependent lung and pulmonary vascular resistance. Furthermore, the absolute values of ITBVI, GEDVI, and EVLWI in this study need to be interpreted with caution, since there were no reference tables and they vary between species (Längin et al., 2020). Additionally, HPV is more pronounced in pigs than in humans and other species (Tucker and Rhodes, 2001). Thus, we could not extrapolate the findings directly to human patients. Second, we addressed only the short-term effects of the different body positions during OLV, although OLV is usually limited to short periods. Third, we did not measure lactate as a surrogate of organ hypoxia. However, we determined mixed venous oxygen saturation, which is an important marker for the oxygen supply of organs (Janotka and Ostadal, 2021).

## Conclusions

During OLV in endotoxemic pigs, the relative perfusion of the ventilated lung and oxygenation were higher in the lateral than the supine position and not impaired by hypovolemia.

## Data Availability Statement

The raw data supporting the conclusions of this article will be made available by the authors, without undue reservation.

## Ethics Statement

The animal study was reviewed and approved by Landesdirektion Sachsen, 09105 Chemnitz.

## Author Contributions

JW, MS, TK, RH, and MG planned and designed the study. JW, MS, XR, DK, ST, RT, and RH conducted the experiments. JW, MS, YC, TB, TK, MS, PR, PP, MG, and RH were involved in the analyses of the data. JW, XR, YZ, DK, ST, RT, YC, JF, SM, and RH cut and soaked the lungs and measured and analyzed fluorescence. JW, MS, MJS, TB, TK, PR, PP, MG, and RH wrote the draft of the manuscript. All authors have read and approved the submitted manuscript, agreed to be accountable for the content of the article, and agreed with its publication.

## Funding

This study was supported by departmental funds.

## Conflict of Interest

MG received consultation fees from Dräger, Ambu, GE Healthcare, and ZOLL. The remaining authors declare that the research was conducted in the absence of any commercial or financial relationships that could be construed as a potential conflict of interest.

## Publisher's Note

All claims expressed in this article are solely those of the authors and do not necessarily represent those of their affiliated organizations, or those of the publisher, the editors and the reviewers. Any product that may be evaluated in this article, or claim that may be made by its manufacturer, is not guaranteed or endorsed by the publisher.

## Abbreviations

CV: Coefficient of variation
EIT: Electrical impedance tomography
E_RS_: Elastance of the respiratory system
EVLWI: Extravascular lung water index
F_I_O_2_: Inspired fraction of oxygen
GEDVI: Global end-diastolic volume index
HPV: Hypoxic pulmonary vasoconstriction
I:E: Inspiratory to expiratory time ratio
ITBVI: Intrathoracic blood volume index
LPS: Lipopolysaccharide
OLV: One-lung ventilation
PEEP: Positive end-expiratory pressure
PICCO: Pulse Contour Cardiac Output
Pmean: Mean airway pressure
Ppeak: Peak airway pressure
PVRI: Pulmonary vascular resistance index
Q_rel, I_: Relative pulmonary blood flow
RR: Respiratory rate
R_RS_: Resistance of the respiratory system
SVRI: Systemic vascular resistance index
TLV: Two-lung ventilation
V_T_: Tidal volume.
