# Supplementary material for: Effects of Body Position and Hypovolemia on the Regional Distribution of Pulmonary Perfusion During One-Lung Ventilation in Endotoxemic Pigs
Source: Front Physiol. 2021 Sep 10;12:717269. doi: 10.3389/fphys.2021.717269 (PMC8461176; doi:10.3389/fphys.2021.717269)
Supplement: Supplementary file 1 [file Data_Sheet_1.docx]

Supplementary Material

## Table 1: Haemodynamic variables

| **Variable** | **Group** | **BL** | **TLVsupine** | | **OLVsupine** | | **OLVsemilateral** | | **OLVlateral** | | **OLVprone** | | **Sequence**  **P=** | **Group**  **P=** | **PositionP=** | **ME**  **P=** |
| --- | --- | --- | --- | --- | --- | --- | --- | --- | --- | --- | --- | --- | --- | --- | --- | --- |
| HR [min^-1^] | Normo | 109 ± 8 | 110 ± 13 | s, se, l, p | 149 ± 26 |  | 146 ± 20 |  | 128 ± 18 | s, se, p | 153 ± 31 |  | 0.286 | 0.220 | ≤0.001 | 0.793 |
|  | Hypo | 111 ± 21 | 126 ± 41 |  | 166 ± 39 |  | 164 ± 34 |  | 154 ± 42 |  | 170 ± 38 |  |  |  |  |  |
| CO  [L min^-1^] | Normo | 5.61 ± 0.76 | 5.57 ± 0.47 | s, se, l, p | 5.81 ± 1.55 |  | 6.61 ± 2.45 |  | 6.6 ± 1.98 |  | 5.82 ± 2.31 |  | 0.281 | 0.640 | 0.008 | 0.270 |
|  | Hypo | 5.96 ± 1.06 | 4.78 ± 1.01 |  | 6.89 ± 1.45 |  | 7.53 ± 2.19 |  | 6.5 ± 1.53 |  | 6.52 ± 3.31 |  |  |  |  |  |
| SV [mL] | Normo | 47 ± 10 | 51 ± 8 | p | 41 ± 14 |  | 43 ± 8 |  | 47 ± 8 |  | 38 ± 11 |  | 0.790 | 0.560 | 0.017 | 0.760 |
|  | Hypo | 60 ± 10 | 46 ± 17 |  | 39 ± 8 |  | 44 ± 8 |  | 41 ± 10 |  | 37 ± 13 |  |  |  |  |  |
| MAP [mmHg] | Normo | 76 ± 13 | 84 ± 8 |  | 81 ± 18 |  | 90 ± 12 |  | 89 ± 16 |  | 82 ± 15 |  | 0.481 | 0.152 | 0.237 | 0.619 |
|  | Hypo | 78 ± 8 | 76 ± 15 |  | 69 ± 18 |  | 78 ± 22 |  | 73 ± 17 |  | 76 ± 19 |  |  |  |  |  |
| MPAP [mmHg] | Normo | 17 ± 4 | 28 ± 10 | s, se, l | 31 ± 8 |  | 35 ± 7 |  | 33 ± 9 |  | 31 ± 5 |  | 0.130 | 0.452 | 0.012 | 0.556 |
|  | Hypo | 17 ± 3 | 26 ± 8 |  | 30 ± 8 |  | 30 ± 9 |  | 31 ± 7 |  | 31 ± 4 |  |  |  |  |  |
| PCWP [mmHg] | Normo | 8 ± 3 | 10 ± 1 | se,le | 8 ± 3 | se, l | 8 ± 2 |  | 6 ± 2 |  | 9 ± 3 | se,, l | 0.777 | 0.111 | ≤0.001 | 0.254 |
|  | Hypo | 7 ± 3 | 6 ± 3 |  | 8 ± 5 |  | 5 ± 2 |  | 5 ± 4 |  | 7 ± 2 |  |  |  |  |  |
| CVP [mmHg] | Normo | 5 ± 2 | 4 ± 2 |  | 3 ± 3 |  | 5 ± 5 |  | 2 ± 3 |  | 1 ± 3 |  | 0.587 | 0.162 | 0.062 | 0.671 |
|  | Hypo | 4 ± 3 | 2 ± 4 |  | 2 ± 3 |  | 1 ± 1 |  | 1 ± 1 |  | 0 ± 4 |  |  |  |  |  |
| PVRI [dyn s cm^-5^  m^-2^] | Normo | 143 ± 58 | 304 ± 192 |  | 387 ± 128 |  | 431 ± 188 |  | 383 ± 105 |  | 382 ± 164 |  | 0.021 | 0.902 | 0.685 | 0.196 |
|  | Hypo | 158 ± 50 | 425 ± 285 |  | 305 ± 111 |  | 344 ± 184 |  | 391 ± 181 |  | 395 ± 151 |  |  |  |  |  |
| SVRI [dyn s cm^-5^  m^-2^] | Normo | 1197 ± 191 | 1710 ± 788 |  | 1473 ± 753 |  | 1300 ± 460 |  | 1326 ± 363 |  | 1469 ± 555 |  | 0.253 | 0.259 | 0.107 | 0.842 |
|  | Hypo | 1110 ± 282 | 1495 ± 263 |  | 1001 ± 371 |  | 1074 ± 408 |  | 1194 ± 548 |  | 1327 ± 597 |  |  |  |  |  |
| EVLWI [mL kg^-1^] | Normo | 9 ± 1 | 8 ± 1 | s, se, l, p | 9 ± 1 |  | 9 ± 2 |  | 9 ± 1 |  | 10 ± 2 |  | 0.147 | 0.077 | 0.006 | 0.731 |
|  | Hypo | 9 ± 1 | 8 ± 1 |  | 10 ± 2 |  | 11 ± 2 |  | 10 ± 1 |  | 11 ± 5 |  |  |  |  |  |
| ITBVI  [mL m^-2^] | Normo | 694 ± 116 | 722 ± 127 |  | 637 ± 200 |  | 670 ± 207 |  | 673 ± 183 |  | 686 ± 249 |  | 0.719 | 0.122 | 0.222 | 0.984 |
|  | Hypo | 765 ± 79 | 619 ± 122 |  | 535 ± 72 |  | 575 ± 122 |  | 560 ± 180 |  | 552 ± 94 |  |  |  |  |  |
| GEDVI  [mL m^-2^] | Normo | 556 ± 93 | 578 ± 102 |  | 510 ± 160 |  | 537 ± 165 |  | 538 ± 146 |  | 549 ± 199 |  | 0.623 | 0.122 | 0.218 | 0.978 |
|  | Hypo | 612 ± 63 | 496 ± 98 |  | 428 ± 58 |  | 461 ± 97 |  | 448 ± 144 |  | 440 ± 76 |  |  |  |  |  |

Mean ± SD; Normo, Normovolemia group; Hypo, hypovolemia group; BL, baseline; TLV, two-lung ventilation; OLV, one-lung ventilation; HR, heart rate; CO, cardiac output; SV, stroke volume; MAP, mean arterial blood pressure; MPAP, mean pulmonary arterial blood pressure; PCWP, pulmonary capillary wedge pressure; CVP, central venous blood pressure; PVRI, pulmonary vascular resistance index; SVRI, systemic vascular resistance index; EVLWI, extra vascular lung water index; ITBVI, intra thoracic blood volume index; GEDVI, global end diastolic volume index; ME, mixed effects position x group. Significance was accepted at P<0.05. Differences between the two groups, the respective body position and sequences of interventions were compared using linear mixed-effects model with repeated measures with TLVsupine, OLVsupine, OLVsemilateral, OLVlateral and OLVprone as within-subject factor and with group and sequence as fixed between subject-factors. The significance of the within-subject factors was corrected for sphericity according to Greenhouse-Geisser. Pairwise post hoc multiple comparisons were performed according to least significant difference (LSD) when appropriate. s P<0.05 vs OLVsupine, se P<0.05 vs OLVsemilateral, l P<0.05 vs OLVlateral, p P<0.05 vs OLVprone.

## Table 2: Respiratory variables

| **Variable** | **Group** | **BL** | **TLVsupine** | | **OLVsupine** | | **OLVsemilateral** | | **OLVlateral** | | **OLVprone** | | **Sequence**  **P=** | **Group**  **P=** | **PositionP=** | **ME**  **P=** |
| --- | --- | --- | --- | --- | --- | --- | --- | --- | --- | --- | --- | --- | --- | --- | --- | --- |
| V_T_  [mL kg^-1^] | Normo | 6.6 ± 0.4 | 6.4 ± 0.1 | s, se, l, p | 5.1 ± 0.2 |  | 5.1 ± 0.2 |  | 5 ± 0.2 |  | 5.1 ± 0.2 |  | 0.446 | 0.866 | ≤0.001 | 0.441 |
|  | Hypo | 6.5 ± 0.1 | 6.4 ± 0.1 |  | 5 ± 0.2 |  | 5.1 ± 0.2 |  | 5.1 ± 0.2 |  | 5.1 ± 0.2 |  |  |  |  |  |
| Ppeak [cmH_2_O] | Normo | 20.7 ± 2.3 | 21.4 ± 3.2 | s, se, l, p | 33.5 ± 5.3 | l | 34.2 ± 2.9 |  | 34.3 ± 3 |  | 34 ± 4.5 |  | 0.485 | 0.995 | ≤0.001 | 0.168 |
|  | Hypo | 19.9 ± 3.2 | 22.2 ± 4.1 |  | 34.8 ± 5.1 |  | 33.1 ± 4 |  | 32.2 ± 4.2 |  | 35 ± 2.9 |  |  |  |  |  |
| Pplat [cmH_2_O] | Normo | 16.7 ± 2 | 18.3 ± 2.6 | s, se, l, p | 28.7 ± 6.3 | l | 29.4 ± 3.7 |  | 29.5 ± 4.5 |  | 29 ± 4.7 |  | 0.463 | 0.897 | ≤0.001 | 0.140 |
|  | Hypo | 16 ± 3.4 | 18.7 ± 4.1 |  | 30.6 ± 5.8 |  | 28.4 ± 4.9 |  | 26.5 ± 4.8 |  | 29.5 ± 4 |  |  |  |  |  |
| Pmean [cmH_2_O] | Normo | 10.6 ± 0.7 | 10.8 ± 1.1 | s, se, l, p | 15.2 ± 1.9 |  | 15.6 ± 1.1 |  | 15.5 ± 1.2 |  | 15.9 ± 2.6 |  | 0.387 | 0.687 | ≤0.001 | 0.312 |
|  | Hypo | 10.4 ± 1 | 11.2 ± 1.3 |  | 15.4 ± 1.6 |  | 15 ± 1.5 |  | 14.6 ± 1.4 |  | 15.6 ± 1 |  |  |  |  |  |
| PEEP [cmH_2_O] | Normo | 5.1 ± 0.1 | 4.9 ± 0.1 |  | 5 ± 0.2 |  | 5 ± 0.1 |  | 5 ± 0.1 |  | 5 ± 0.1 |  | 0995 | 0.267 | 0.422 | 0.142 |
|  | Hypo | 5.1 ± 0.1 | 5 ± 0.2 |  | 4.9 ± 0.2 |  | 5 ± 0.1 |  | 4.9 ± 0.2 |  | 4.8 ± 0.3 |  |  |  |  |  |
| RR [min^-1^] | Normo | 30 ± 3 | 29 ± 2 | s, se, l, p | 35 ± 0 |  | 35 ± 2 |  | 35 ± 0 |  | 35 ± 0 |  | 0.376 | 0.854 | ≤0.001 | 0.531 |
|  | Hypo | 30 ± 0 | 29 ± 2 |  | 35 ± 0 |  | 35 ± 0 |  | 35 ± 0 |  | 35 ± 0 |  |  |  |  |  |
| MV  [L min^-1^] | Normo | 8.7 ± 0.8 | 8.2 ± 0.4 | s, se, l, p | 7.8 ± 0.5 |  | 7.6 ± 0.3 |  | 7.7 ± 0.5 |  | 7.8 ± 0.5 |  | 0.982 | 0.731 | 0.05 | 0.747 |
|  | Hypo | 8.4 ± 0.9 | 8.1 ± 0.1 |  | 7.7 ± 0.8 |  | 7.7 ± 0.9 |  | 7.8 ± 0.1 |  | 7.7 ± 0.1 |  |  |  |  |  |
| E_RS_ [cmH_2_O L^-1^] | Normo | 35.4 ± 6.8 | 42.0 ± 11.1 |  | 83.5 ± 26.5 |  | 86.1 ± 15.2 |  | 90.3 ± 17.3 |  | 87.1 ± 22.3 |  | 0.423 | 0.807 | ≤0.001 | 0.124 |
|  | Hypo | 33.8 ± 10.3 | 43.3 ± 9.1 |  | 92.9 ± 19.6 |  | 83.5 ± 13.0 |  | 76.7 ± 11.9 |  | 85.4 ± 11.0 |  |  |  |  |  |
| R_RS_ [cmH_2_O s L^-1^] | Normo | 11.1 ± 1.3 | 11.7 ± 1.8 |  | 24.9 ± 5.4 |  | 25.9 ± 3.8 |  | 24.9 ± 2.6 |  | 25.3 ± 5.2 |  | 0.395 | 0.792 | ≤0.001 | 0.565 |
|  | Hypo | 11.8 ± 1.4 | 11.7 ± 1.6 |  | 24.3 ± 4.1 |  | 24.4 ± 3.8 |  | 23.6 ± 2.7 |  | 27.1 ± 6.3 |  |  |  |  |  |

Mean ± SD; Normo, Normovolemia group; Hypo, hypovolemia group; BL, baseline; TLV, two-lung ventilation; OLV, one-lung ventilation; V_T_, tidal volume; Ppeak, peak airway pressure, Pplat, plateau airway pressure, Pmean, mean airway pressure; PEEP, positive end expiratory pressure; RR, respiratory rate, MV, minute volume; E_RS_, elastance of the respiratory system, R_RS_, resistance of the respiratory system; ME, mixed effects position x group. Significance was accepted at P<0.05. Differences between the two groups, the respective body position and sequences of interventions were compared using linear mixed-effects model with repeated measures with TLVsupine, OLVsupine, OLVsemilateral, OLVlateral and OLVprone as within-subject factor and with group and sequence as fixed between subject-factors. The significance of the within-subject factors was corrected for sphericity according to Greenhouse-Geisser. Pairwise post hoc multiple comparisons were performed according to least significant difference (LSD) when appropriate. s P<0.05 vs OLVsupine, se P<0.05 vs OLVsemilateral, l P<0.05 vs OLVlateral, p P<0.05 vs OLVprone.
